# Quantitative Imaging of Single Upconversion Nanoparticles in Biological Tissue

**DOI:** 10.1371/journal.pone.0063292

**Published:** 2013-05-14

**Authors:** Annemarie Nadort, Varun K. A. Sreenivasan, Zhen Song, Ekaterina A. Grebenik, Andrei V. Nechaev, Vladimir A. Semchishen, Vladislav Y. Panchenko, Andrei V. Zvyagin

**Affiliations:** 1 MQ Biofocus Research Centre, Macquarie University, Sydney, NSW, Australia; 2 Department of Biomedical Engineering and Physics, Academic Medical Center, University of Amsterdam, Amsterdam, The Netherlands; 3 Shemyakin-Ovchinnikov Institute of Bioorganic Chemistry, Russian Academy of Sciences, Moscow, Russia; 4 HTBAS Department, Lomonosov Moscow State University of Fine Chemical Technologies, Moscow, Russia; 5 Institute of Laser and Information Technology, Russian Academy of Sciences, Troitsk, Moscow Region, Russia; King Abdullah University of Science and Technology, Saudi Arabia

## Abstract

The unique luminescent properties of new-generation synthetic nanomaterials, upconversion nanoparticles (UCNPs), enabled high-contrast optical biomedical imaging by suppressing the crowded background of biological tissue autofluorescence and evading high tissue absorption. This raised high expectations on the UCNP utilities for intracellular and deep tissue imaging, such as whole animal imaging. At the same time, the critical nonlinear dependence of the UCNP luminescence on the excitation intensity results in dramatic signal reduction at (∼1 cm) depth in biological tissue. Here, we report on the experimental and theoretical investigation of this trade-off aiming at the identification of optimal application niches of UCNPs e.g. biological liquids and subsurface tissue layers. As an example of such applications, we report on single UCNP imaging through a layer of hemolyzed blood. To extend this result towards *in vivo* applications, we quantified the optical properties of single UCNPs and theoretically analyzed the prospects of single-particle detectability in live scattering and absorbing bio-tissue using a human skin model. The model predicts that a single 70-nm UCNP would be detectable at skin depths up to 400 µm, unlike a hardly detectable single fluorescent (fluorescein) dye molecule. UCNP-assisted imaging in the ballistic regime thus allows for excellent applications niches, where high sensitivity is the key requirement.

## Introduction

Optical imaging of biological tissues provides highly informative, non-invasive and inexpensive means to assess the tissue physiological status and functionality, especially for diagnosis of pathological sites. Labeling these tissue sites with luminescent biocomplexes, often referred to as molecular probes, improves localization accuracy and sensitivity. Deployment of a new class of molecular probes whose excitation/emission falls into the so-called biological tissue transparency window (650 nm –1300 nm) allows deeper imaging in virtue of the minimized absorption and scattering of biotissue in this near-infra-red (NIR) wavelength range [1], [2]. For example, specific labeling of cancerous lesions with a molecular probe based on indocyanine green (the only NIR organic dye approved for routine clinical procedures) provided up to a 4-fold increase in the tumor imaging contrast in comparison with that achieved using the intrinsic hemoglobin spectral signature and tissue scattering [3], [4].

The development of NIR-emitting probes has traditionally been directed towards organic dyes, but more recent studies have demonstrated considerable promise of inorganic nanoparticle-based probes, such as quantum dots (QDs) and most novel upconversion nanoparticles (UCNPs) [5], [6]. Organic NIR-dyes are favorably small, easily dispersed in an aqueous environment and are amenable to bioconjugation utilizing established protocols. However, their poor thermal and photochemical stability (photobleaching) and a low fluorescence quantum yield (QY) (5–25% in NIR, with propensity to deteriorate in biological environments) are inferior in comparison with QDs whose attractive optical properties include size-tunable optical absorption and emission spectra, high photochemical stability, a large QY (20–70% in NIR), and high thermal stability [7]. On the other hand, QD fluorescence is intermittent (‘blinking’) hindering applications such as single molecule tracking, while QD intrinsic toxicity largely precludes their use *in vivo* [8]. UCNPs share the high photochemical and thermal stability of QDs and other inorganic nanomaterials, complemented with non-blinking emission and demonstrated biocompatibility. In addition, UCNPs can be employed as a docking platform for high drug payloads for targeted delivery [9]. The key advantage of UCNPs is their unique photochemical structure that enables “upconversion” of NIR excitation light (980 nm) of modest intensity (100 W/cm^2^) to the higher energy visible emission (450–850 nm) [10]. Since no known biological molecule is capable of such conversion [1], [11], the intrinsic tissue fluorescence, termed autofluorescence, can be eliminated in the detection path by conventional optical short-pass filtering. In addition, the exceptionally long (sub-ms) luminescence lifetimes of UCNPs allow realization of time-gated detection schemes that can completely suppress the residual back-scattered excitation light bleeding through the (interference) spectral filters [12]. Since *in vivo* imaging performance is crucially dependent on the contrast provided by the molecular probe [1], background-free detection of UCNPs is very promising, as has been shown by the autofluorescence-free trans-illumination imaging in mice using biocompatible UCNPs [13], [14].

The unique upconversion property of UCNPs is a result of the sequential photon absorption and energy transfer processes within an inorganic host matrix, with hexagonal-phase β-NaYF_4_ co-doped with trivalent lanthanide ions reported to be the most efficient [15], [16]. These dopant ions are classified as sensitizers and activators considering their respective roles in the UCNP absorption and emission. The sensitizer, typically Ytterbium ion (Yb^3+^), absorbs the NIR-radiation energy and transfers it non-radiatively to the closely spaced neighboring Yb^3+^, forming a network of excited Yb^3+^ (referred to as delocalized quasi-exciton [17]) until the energy is seized by activator ions, usually Erbium (Er^3+^), or Thulium (Tm^3+^). The activator makes a transition to a metastable excited state, from where it can coalesce with a nearby excited Yb^3+^-ion, to be transferred to the next energy level (a process called energy transfer upconversion) at the expense of the participating Yb^3+^ decaying to the ground state. Multiple-step energy transfer upconversion is also possible. The activator can return to the ground state by radiating a photon in the visible or NIR wavelength range. The upconversion emission results from the absorption of 2 or more NIR photons, and hence exhibits a supralinear dependence on the excitation intensity, *I*_ex_, as quantified by the conversion efficiency (*η*_uc_) addressed in Section 2.1.2. In contrast with other anti-Stokes processes occurring at *I*_ex_ ∼ 1×10^5^ W/cm^2^ [18], the energy transfer upconversion occurs via a real metastable excited state(s) at rather modest *I*_ex_ ∼ 1×10^2^ W/cm^2^ that is readily achievable by focusing a continuous-wave excitation beam.

The specific photophysical properties of upconversion nanoparticles entail several challenges for optical biomedical imaging. Firstly, the quasi-excitonic nature of UCNP excitation renders the emission size dependent, susceptible to surface quenching [19] and vulnerable to (biological) environment, which causes reduction of *η*_uc_ values that are generally small compared to these of QDs or organic fluorescent dyes. Values of *η*_uc_ are typically around 1% for nanoparticle diameters of ∼50 nm, shown to be the optimal size for cellular receptor-mediated internalization [20]. Secondly, *η*_uc_ is dependent on the excitation intensity and increases to a plateau value, for which the *I*_ex_ is referred to as saturation intensity, *I*_sat_ (*I*_sat_ 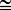
1×10^2^ W/cm^2^). Keeping *I*_ex_ close to *I*_sat_ is preferable for biomedical imaging.

This dependency of *η*_uc_ on *I*_ex_ poses the main challenge for UCNP-assisted optical imaging in deep (live) tissue layers exceeding centimeter(s). Indeed, the attainment of *I*_sat_ by focusing is hardly possible in turbid biotissue, while the laser power is limited by the allowed maximum permissible exposures (MPEs). The UCNP luminescence signal is therefore diminished with depth as was demonstrated in tissue phantom experiments [14]. In another recent study, the critical dependence of *η*_uc_ versus depth was manifested by the considerable deterioration of axial and lateral resolution of full-field upconversion microscopy at depths of ∼300–400 µm [21]. These results urge identification of practical application niches for UCNPs that will benefit from the afore-mentioned advantages of UCNPs, while not being restricted by their limitations. Promising scenarios include UCNP-imaging in biological fluids, thick slices and subsurface tissue layers, where the UCNP based molecular probe contrast is expected to be superior to that of existing molecular probes, including organic fluorescent dyes.

This work aims to explore the cutting-edge optical imaging scenario represented by a single UCNP buried in an absorbing biological environment, which was demonstrated by single-UCNP imaging through a layer of hemolyzed blood. We believe this result has not been reported before [22]–[25]. We report on absolute conversion efficiency and spectral properties of single UCNPs. The subsequent thorough characterization of the UCNP emission signal versus the excitation/detection parameters allowed projection of the obtained experimental results onto a theoretical model, generalized to biomedical imaging applications of extreme sensitivity in challenging *in vivo* environments. Single-UCNP imaging in live skin was modeled to be feasible in skin at depths up to a few hundred micrometers, with superior contrast compared to a conventional fluorescent dye molecule. The results show that imaging in the ballistic regime allows for excellent applications of UCNP-guided imaging in life sciences where high sensitivity is a key requirement.

## Materials and Methods

### 2.1 Synthesis

#### Synthesis reagents

Y_2_O_3_, Yb_2_O_3_, Er_2_O_3_, sodium trifluoroacetate 99%, trifluoroacetic acid 98%, oleic acid 90%, 1-octadecene 90% (all purchased from Sigma-Aldrich).

#### Synthesis protocol of β-NaYF_4_:Yb,Er nanophosphors

A mixture of Y_2_O_3_ (0.78 mmol), Yb_2_O_3_ (0.20 mmol), Er_2_O_3_ (0.02 mmol) was suspended in 70% trifluoroacetic acid (20 ml) and gently refluxed until a clear solution was obtained (≈ 6 h), then cooled to room temperature. After the solution was evaporated, the formed residue was dried in vacuum (0.1 torr, 3 h). The resulting slurry was thoroughly ground in agate mortar to obtain a fine homogenous powder. This rare-earth trifluoroacetate mixture, together with sodium trifluoroacetate (1 mmol), was added to oleic acid (6 ml) and 1-octadecene (6 ml) in a three-neck flask equipped with a thermometer, septum stopper and glass magnetic stirrer. Next, the solution was heated to 100°C and stirred under vacuum for 30 min for degassing and removal of water. The mixture was subsequently gradually heated at a rate of 8°C/min to 290°C, and maintained at this temperature for 45 min under argon atmosphere. Next, a solution of sodium trifluoroacetate (1 mmol) in oleic acid (2 ml) and of 1-octadecene (2 ml), heated to 85°C, was added to the reaction, after which the reaction temperature was raised to 330°C and stirred for 15 min under argon. Next, isopropanol (130 ml) was added to the cooled solution and the mixture was centrifuged at 6000 rpm for 30 min. The resultant nanoparticles were washed with absolute ethanol (4 times), dried, dissolved in chloroform (10 ml), precipitated with isopropanol (50 ml) and centrifuged at 4000 rpm for 10 min. The last procedure was repeated 2 times. The residue was dried at room temperature.

### 2.2 Imaging

#### 2.2.1 Transmission electron microscopy

The UCNPs were dissolved in hexane, drop-casted on Formav©-coated TEM grids and dried in a desiccator at room temperature. The grids were imaged with a Philips CM10 TEM and analyzed using ImageJ free-ware to obtain the UCNP size distributions. For single-UCNP imaging, the hexane solutions were diluted and drop-casted on Formav©-coated TEM nickel finder grids for easy navigation. The thinly coated finder grids can be imaged using TEM and optical epi-luminescence systems. In order to find single nanoparticles, low-magnification TEM imaging was carried out gradually zooming into areas of interest to track the UCNP location precisely.

#### 2.2.2 Laser-illuminated inverted epi-luminescence microscope

**Figure 1:**
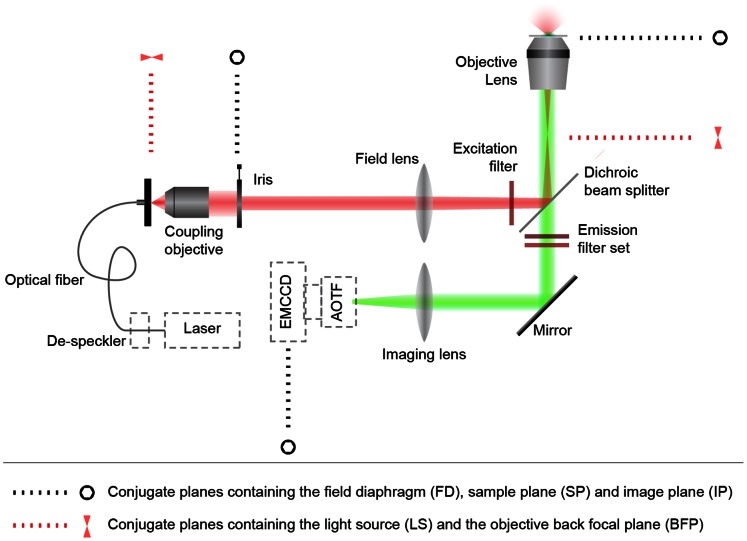
Diagram of the custom-modified epi-luminescence imaging system employed for single-UCNP and spectral imaging. A wide-field inverted epi-luminescence microscope was modified to allow external fiber-coupled laser illumination. The optical fiber was dithered to average out speckles. The excitation light was configured to uniformly illuminate the field-of-view at the sample plane via a modified Köhler illumination scheme. The sample plane was imaged using an EMCCD camera, optionally mounted with an AOTF for hyperspectral imaging. An adjustable iris diaphragm allowed reduction of the field-of-view to restrict imaging to several single UCNP particles and small clusters.

A wide-field inverted epi-luminescence microscope (Olympus IX70) equipped with a water-immersion objective (40×, NA 1.15, Olympus) was modified to allow external laser illumination at the sample plane (fiber-coupled diode laser at wavelength 978 nm, LD980-01CW CXCH-Photonics). In order to achieve uniform illumination of the size-controllable field-of-view at the sample plane, a modified Köhler illumination scheme was designed and built in-house, as shown in Figure 1, with detailed description and ray diagram provided in Figure S1. In brief, an image of the field diaphragm placed in the excitation beam path was formed at the sample plane (conjugate planes FD and SP, Figure 1), while the excitation beam was homogenized and collimated (owing to the conjugate planes LS and BFP, Figure 1). In order to reduce laser speckle induced sample illumination non-uniformity, the laser-coupling fiber was mechanically dithered at a frequency >5 KHz. A zero-aperture adjustable iris diaphragm served as the field diaphragm allowing tailoring of the field-of-view at the sample plane, to a diameter as small as 20 µm that contained only several UCNPs. The sample image was captured by an electron-multiplying CCD (EMCCD) camera (Andor iXon DU-885) mounted to the microscope detection port. An acousto-optic tunable filter (AOTF) (LSi-300 Hyperspectral Imaging System, Gooch and Housego) was integrated into the detection path of the epi-luminescence microscope for hyperspectral imaging. The employed spectral range was 500–700 nm with 3-nm increments and a bandwidth of 3.7 nm. The spectral filter module (filter cube) contained a high-pass absorbance filter (cut-off 850 nm, Thorlabs) placed in the excitation beam path to remove the 978-nm laser side lobs. A dichroic beam-splitter (cut-off, 511 nm, Semrock) reflected the excitation light toward the sample, while passing the emitted light to the detection path. Two additional short-pass filters (cut-off, 842 nm, Semrock and cut-off, 700 nm, Thorlabs) in the detection channel suppressed the excitation light leakage (see Figure S2). At the sample plane, the TEM finder grid with UCNPs was placed on a microscope glass slide and covered with a standard cover slip.

### 2.3 Emission Spectra (Ensemble)

The UCNP powder was placed in a custom-designed sample holder that consisted of a thin glass plate (thickness, 0.45 mm) with a 1.5-mm diameter circular hole in the center sandwiched between two glass cover-slips. The excitation fiber was butted against the glass facing the hole filled with the UCNP powder. The emission spectrum was measured in transmission using a calibrated fiber-coupled diffraction-grating based spectrometer (Ocean Optics) in conjunction with a short-pass emission filter (cut-off, 842 nm).

### 2.4 Conversion Efficiency

#### 2.4.1 Absolute conversion efficiency of UCNPs (ensemble)

The UCNP powder was placed in the custom-designed sample holder (see section 2.3) at one of the exit ports of a 4-inch integrating sphere (Labsphere), as described by Page *et al* [15]. At the backside of the sample holder, a tilted aluminum-coated glass slide was placed to reflect the light back into the integrating sphere avoiding a double-pass through the sample. A multimode optical illumination fiber (fiber core 400 µm, NA 0.22) was butted against the sample holder at the center of the hole. This assured precise control and reproducibility of the excitation spot size at the UCNP sample to calculate *I*_ex_. Luminescence emitted by the UCNP sample was spatially integrated in the sphere by means of multiple reflections from the walls, and eventually detected using a photodiode (PD) (Thorlabs, PDA-55) at the exit port positioned at 90° with respect to the illumination path. Using a lock-in amplifier (Stanford Research Systems, model SR830) connected to the PD output and pulsed laser excitation (laser: LD980-01CW CXCH-Photonics; and pulse generator: Stanford research systems, model DG535, pulse width 4 ms, frequency 125 Hz) enabled reliable registration of the UCNP luminescence signals as low as 1 µW. The *I*_ex_ range was controlled within several decades by means of the laser diode current and neutral density filters. The UCNP emission and unabsorbed excitation powers were measured by inserting a short-pass (cut-off, 842 nm) and long-pass (cut-off, 830 nm, Semrock) filter, respectively, in the detection channel (in front of the PD). The performance of the interference filters and the detector channel collection efficiency were optimized by careful positioning a high-power lens in the detection channel. The spectral response of the integrating sphere and PD was calibrated for a broad spectral range (470–1050 nm). By definition, *η*_uc_ was calculated as *P*_em_ */P*_abs_ [W/W]. *P*_em_ was determined by the straightforward measurement of the luminescence signal corrected for the absolute spectral response of the system and the UCNP spectral emission (see Figure S3A). *P*_abs_ was determined by measuring the excitation powers unabsorbed by a reference sample (TiO_2_), *P*_ref_, and the UCNP powder, *P*_UCNP_, so that *P*_abs_ = *P*_ref_ *– P*_UCNP_. *P*_abs_ mainly resulted from the linear absorption of Ytterbion ions. For robust estimation of *P*_abs_, the measurements were repeated for several excitation intensities and a linear fit was performed to calculate the absorbed fraction of the excitation light (r^2^>0.99).

#### 2.4.2. Absolute conversion efficiency of single-UCNP

UCNPs sparsely deposited on the TEM finder grid were imaged using the epi-luminescence microscope set-up, as described earlier. The emitted power was determined by reading out the pixel values of the EMCCD image using the camera settings, sensitivity specifications and by calibrating the throughput of the microscope optics from sample plane to image plane. Two aspects of the EMCCD related to the epi-luminescence imaging are important to note: 1. The sensor quantum efficiency, *QE*_CCD_(*λ*) [e^-^ per photon] is wavelength-dependent, so that integration over the relevant wavelength range (400–800 nm) was necessary. 2. The camera electron-multiplication gain (*EM gain*) allowed straightforward multiplication of the pixel read-out values by the *EM gain* (in virtue of RealGain™ EMCCD feature). The UCNP signal read out by the sensor, *S*_UCNP_, depended on the UCNP sample emitted intensity and the spectral calibration coefficient of the imaging system according to:
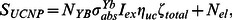
(1)where *S*_UCNP_ and *N*_el_ [counts/s] were the signal per UCNP node and electronic noise level, respectively; *N*_YB_ was the number of Ytterbium (Yb^3+^) ions per node that absorbed the excitation light; *σ*_abs_[cm^2^] was the Yb^3+^ absorption cross-section (neglecting the absorption due to ∼10-fold fewer Er^3+^ ions and 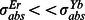) [15], [26]; and *ζ*_total_ [counts/W/s] was the calibration coefficient integrated over the UCNP emission spectrum (see Figure S4). *S*_UCNP_ was the sum of the pixels that sampled an image of the UCNP node. The noise contained electronic dark and read noise components. Optical background was negligible, as shown in Figure S2. The number of Yb^3+^ per UCNP node was estimated using the NaYF_4_ crystal lattice constants (*a* = 5.991 Å, *c* = 3.526 Å) [27], TEM-derived dimensions and numbers, and Na to Yb molar ratio [28].

*ζ*_total_ was calculated according to:
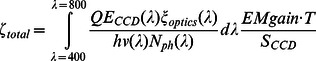
(2)where *ξ*_optics_ is the throughput of the imaging optics, *h* is Planck’s constant, *ν* the light frequency, *N*_ph_ the number of photons per wavelength per UCNP emission power, *T* the exposure time [s], *EMgain* is a linear factor and *S*_CCD_ is the camera sensitivity [e^-^ per count]. The values of *QE*_CCD_ and *S*_CCD_ were provided by the EMCCD manufacturer. The absolute throughput *ξ*_optics_ was spectrally calibrated by imaging of an optical fiber in the sample plane and comparing the known fiber output power to the power detected by the camera at the relevant wavelength range (see Figure S5). In SI (Figure S4), the spectral output and the detector response are shown for UCNP imaging using a water- and blood-immersion objective.

## Results and Discussion

Realization of the prime goal of ultrahigh-sensitivity imaging of UCNPs in biological tissue is critically dependent on the attainable UCNP contrast, which is defined as the ratio of the detected luminescence signal originating from the UCNPs (*S*) to the background signal stemming from the residual biological tissue autofluorescence and noise (*B*). The signal estimation calls for a thorough characterization of the excitation and emission properties of UCNPs, as well as quantification of the excitation/detection paths of the optical microscopy system adapted for ultrahigh-sensitivity imaging, where the adverse effects of the biological tissue on UCNP excitation and detection are taken into account. These effects are greatly exacerbated in the case of most fluorescent organic dyes, as will be shown in our cross-comparison study of optical imaging of a UCNP and fluorescein dye molecule in biological tissue. The background signal estimation demands consideration of the tissue optical properties, including scattering, absorption and autofluorescence. We commence by reporting on the measurement of absolute conversion efficiency and emission spectra of UCNPs, using an integrating sphere set-up (Section 3.1.2; ensemble measurements) and calibrated optical microscopy system (Section 3.1.3 and 3.1.4; single-particle measurements). In Section 3.2 the quantitative assessment of the single-UCNP imaging contrast in biological environment is addressed experimentally using hemolzyed blood and theoretically using a skin model. We combine the physical characteristics of UCNPs, the optical properties of biological tissue and the abilities of advanced imaging systems to provide clear guidance towards intelligent development and a realistic application scope of upconversion nanomaterials.

### 3.1 Quantitative Characterization

#### 3.1.1 Conversion efficiency. Comments on conventions

Conversion efficiency, *η*_uc_ of UCNPs represents the most important parameter that governs their luminescence properties. It is defined as emitted power/absorbed power (*P*_em_ */P*_abs_) expressed in W/W [15]. We note that this important parameter is used inconsistently across the literature. Traditionally, the term “quantum yield” (QY) is defined as the ratio of the number of photons emitted to the number of photons absorbed by the sample [29]. However, since during the upconversion process one photon is emitted as a result of the absorption of two or more photons, the QY would not exceed 50% following this definition. In order to re-normalize this value, the QY of 2- or 3- photon absorption processes is scaled by a factor 2 or 3 respectively [16], [30], although not consistently in literature [13], [31]. In addition, the emission is result of a complex, unknown combination of 2- and 3-photon excitation pathways [32], [33], rendering this approach speculative. For these reasons, the term “conversion efficiency” is believed to more adequately represent the net output of the complex upconversion process.

It is worthwhile to comment here on two other issues with UCNP conventions. Firstly, the dependency of *η*_uc_ on *I*_ex_ deteriorates the comparison of *η*_uc_ or QY measurements taken at different values of *I*_ex_. For example, the reported QY values of 0.18% [22] and 0.47% [13] measured at *I*_ex_ ∼ 10^3^ W/cm^2^ and 17.5 W/cm^2^ respectively, are hardly comparable due to an unknown functional dependence of *η*_uc_ on *I*_ex_. Secondly, quantitative reports on the absolute *η*_uc_ or related values are still scarce in literature [15], [31] often replaced by the limited-accuracy comparisons of UCNP emission with that of bulk phosphors or organic dyes [6], [23]. In order to address this lacuna, we present the results of the measurements of absolute *η*_uc_ of UCNPs synthesized in-house for a large range of excitation intensities up to *I*_sat_.

#### 3.1.2 Photophysical properties of as-synthesized upconversion nanoparticles

**Figure 2:**
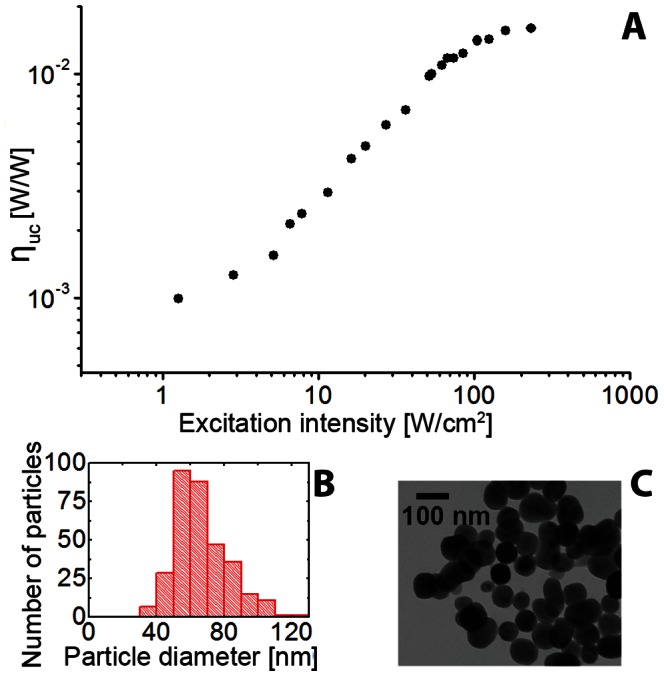
Conversion efficiency, size and morphology of UCNPs synthesized in-house. (A) Plot of the absolute conversion efficiency (*η*_uc_) [W/W] of the reported upconversion nanoparticle sample versus the excitation intensity at *λ*_ex_ = 978 nm measured using a calibrated integrating sphere set-up. *η*_uc_ is the ratio of the emitted power integrated over the entire emission spectral range (500–700 nm) to the absorbed power. (B) Size histogram obtained by analyzing the transmission electron microscopy (TEM) images of NaYF_4_:Yb,Er UCNPs (330 particles). A typical TEM-image is shown in (C).

We carried out characterization of the key photophysical properties of as-synthesized UCNPs in powder form. The β-NaYF_4_ nanocrystals were synthesized following the thermal decomposition method, and co-doped with Yb^3+^ and Er^3+^ ions (β-NaYF_4_:Yb,Er) at the most efficient molar ratio of 20% Yb^3+^ and 2% Er^3+^ [5], [28], [32]. The measurement of the absolute conversion efficiency versus excitation intensity at an excitation wavelength (*λ*_ex_) of 978 nm, was performed using a custom-modified integrating sphere set up. The integrating sphere ensured absolute measurement of the absorption and emission characteristics of UCNP powder independent of scattering by the sample. Size, morphology and *η*_uc_ results are summarized in Figure 2 and full scale TEM images at different magnification levels are provided in Figure S6. The synthesis yielded quasi-spherical UCNPs of diameters measured to be 68±16 nm. The *η*_uc_ was measured to reach nearly 2% at *I*_ex_ ≡ *I*_sat_ ≌ 150 W/cm^2^, a high value in comparison with that of the comparably sized β-NaYF_4_:Yb,Er sample reported by Boyer et al. [31], (QY ≈ 0.3% at comparable *I*_ex_, recalculated using the emission spectrum and photon energies to *η*_uc_ ≈ 0.5%), although the sample environment (organic solvent hexane known to quench *η*_uc_) might well account for this difference. As was expected for the supralinear upconversion process, the *η*_uc_ dropped dramatically at low *I*_ex_, as can be seen in Figure 2A. The UCNP emission spectra versus *I*_ex_ were acquired using a calibrated spectrometer and presented in Figure S3. These spectra exhibited two characteristic emission bands grouped in green (510–560 nm) and red (640–680 nm) emission multiplets, as shown in Figure S3A. The red and green emission bands were attributed to the Er atomic transitions induced by the sequential two and three photon energy absorption processes [10]. The complex multi-step excitation process of the UCNPs is nonlinear, as manifested by the supralinear dependence of the emitted luminescence power, *P*_em_, versus *I*_ex_: 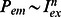, where the power index *n* varies versus *I*_ex_ reaching *n* = 1 at *I*_ex_ = *I*_sat_. At low *I*_ex_, *n* would ideally take discrete values of 2 or 3, reflecting the 2 or 3- step excitation process. However, the measured *n* takes the values of 1.5 and 1.9 for the green and red emission bands respectively (Figure S3C), reflecting more complex processes such as linear decay, exited state absorption, energy transfer upconversion, cross-relaxation and quenching, and their interrelations in the different upconversion pathways [16], [28], [33]–[35]. It is beyond the scope of this paper to elucidate these mechanisms. However, it is important to account for the spectral and *η*_uc_ dependence on the excitation intensity in a quantitative analysis of the UCNP imaging *in vivo.*

The complexity of the upconversion excitation/emission process is confounded by ensemble averaging, where the surface-related (non-radiative relaxation) processes can be concealed by inter-particle interactions. Characterization of an isolated individual UCNP is instrumental to remove this uncertainty.

#### 3.1.3 Single-particle spectral imaging

**Figure 3:**
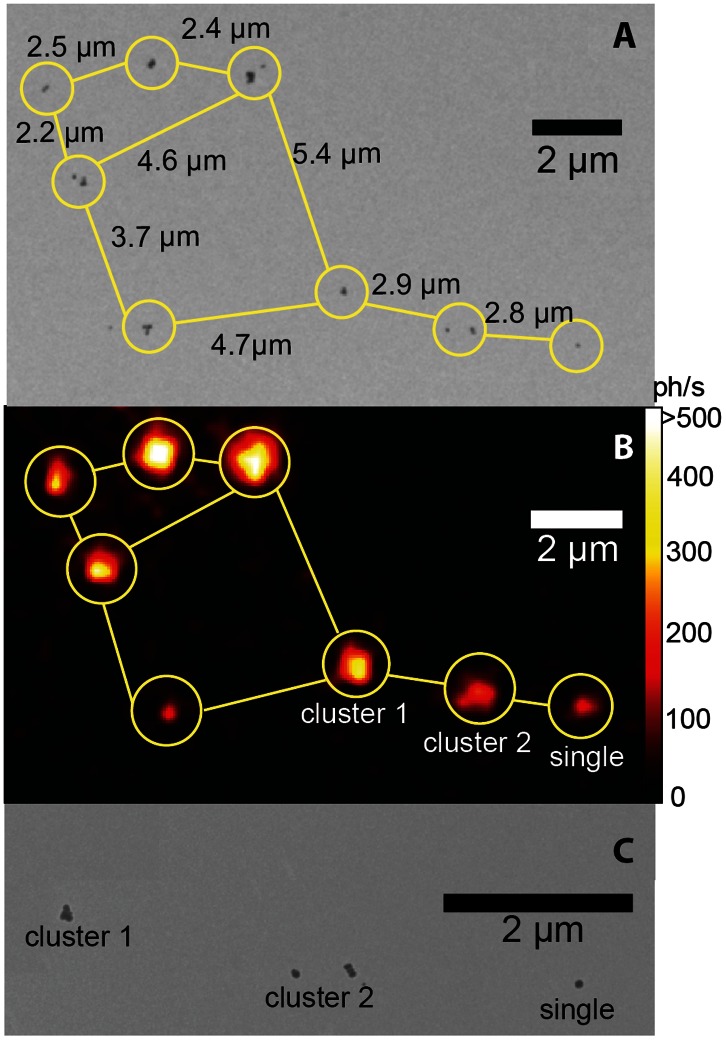
Single-UCNP correlative imaging. (A) TEM and (B) epi-luminescence microscopy images corresponding to the same areas of the sample TEM grid. The distances between the individual (encircled) nanoparticles/clusters, given in (A), were precisely matched to those in (B) to identify the same UCNP constellation. (C) Close-up TEM image of the same area as in (A), where UCNP sites designated ‘cluster 1’, ‘cluster 2’, and ‘single’ correspond to the three sites in (B). The individual UCNPs within ‘cluster 1’ and ‘cluster 2’ were optically unresolvable. “Single” designates a single UCNP particle clearly observable, as a diffraction-limited spot in (B). The excitation wavelength, intensity and exposure time were 978 nm, ∼250 W/cm^2^ and 0.7 s, respectively. The pixel values were converted to photons/second (ph/s) and color-coded according to the look-up color bar in (B).

A single upconversion nanoparticle represents an excellent entity for the quantitative measurements of the emission spectra and *η*_uc_, because it is unaffected by inter-particle interactions and has known physical dimensions. In order to establish this single-UCNP experimental model, we modified a wide-field epi-luminescence inverted microscope to allow uniform illumination of the sample plane with an external excitation laser (978 nm). A high-sensitivity EMCCD camera was incorporated in the microscope detection path. This imaging modality conferred several advantages, including short acquisition times (∼1 s) at moderate *I*_ex_ ≌ 250 W/cm^2^ uniformly distributed across the field-of-view, as compared to flying-spot configurations that operate at *I*_ex_ ≌10^6^ W/cm^2^ and with a pixel dwell time of 10 ms [22], [24], resulting in acquisition times of 40 minutes for a 512×512 pixel image. In order to demonstrate single-UCNP imaging, the as-synthesized powder was dispersed in organic solvent and sparsely deposited on a TEM grid pre-coated with a Formav© monolayer film. This enabled TEM and epi-luminescence imaging of the same sample areas to be matched and individual nanoparticles singled out. An example of this correlative imaging method is shown in Figure 3, where the two top panels display the matched constellation of UCNPs acquired by TEM and epi-luminescence microscopy, respectively, and a single particle (designated “single”) is observable.

**Figure 4:**
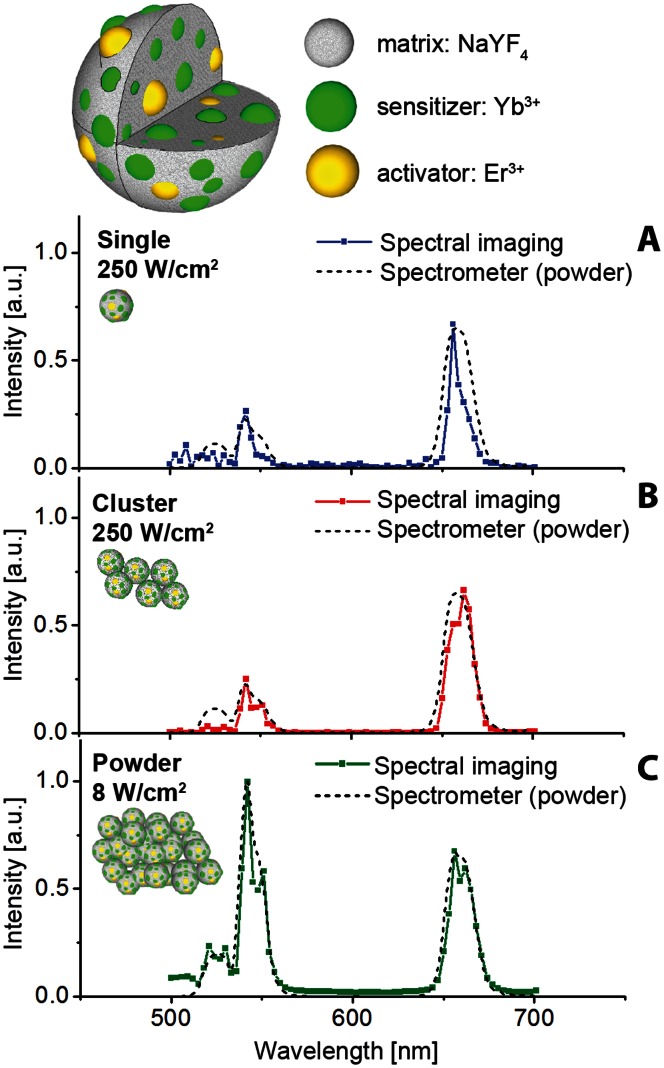
Spectral imaging of UCNPs. Emission spectra of UCNPs in (A) single, (B) small cluster (designated ‘cluster 2’ in Figure 2) and (C) powder form (data points joined by solid lines) captured using hyperspectral epi-luminescence microscopy, overlaid with the ensemble-averaged spectra of UCNP powder captured by a calibrated spectrometer (dashed lines). The corresponding exposure times and EMCCD camera electron-multiplication (EM) gains were (A) 4 sec and 255; (B) 1.5 sec and 44; and (C) 0.014 sec and 9. Since the samples (A) and (B) contained considerably less emitters than the powder sample (C), the excitation intensities at λ_ex_ = 978 nm were varied, respectively, from 250 W/cm^2^ to 8 W/cm^2^ to accommodate for the large disparity in the emission signals that would otherwise exceed the dynamic range of the EMCCD. The decreased I_ex_ resulted in an increased green-to-red emission ratio in (C) due to the varied upconversion energy redistribution between the green and red multiplets. Top panel, schematic diagram of NaYF_4_:Yb,Er UCNP.

The utility of this single-UCNP imaging system is demonstrated by comparison of the luminescence spectra of single, clustered, and powder UCNP samples that were obtained using an acousto-optic tunable filter (AOTF) integrated into the detection path of the epi-luminescence microscope. This enabled acquisition of spectral data from every pixel, converting our microscope into a hyperspectral imaging modality. The spectral responses of “single” and “cluster 2” were examined individually and locally by reading out spectral data from the corresponding pixels (Figures 4A, 3B). The emission spectra of the other small clusters were similar (data not shown). The ensemble-averaged spectrum of the UCNP powder was also obtained using the hyperspectral imaging mode (Figure 4C) by processing a full-field image of the powder sample. In order to cross-validate the spectral measurements, the UCNP powder emission spectra were additionally acquired with a diffraction-grating based calibrated spectrometer (dashed curves, Figure 4). The dependency of the spectral features on excitation intensity is discussed in Section 3.1.2 and Figure S3. The spectral emissions of the individual, clustered and ensemble UCNPs showed a high resemblance at equivalent *I*_ex_. Hence, the UCNP spectral luminescence profile appeared to be primarily dependent on *I*_ex_ and independent on the inter-particle interactions in air, thus clarifying the speculations made by Wu et al. [24] This enabled accurate characterization of *η*_uc_ of single UCNPs, as described in Section 3.1.4.

#### 3.1.4. Measurement of single-particle conversion efficiency

Each node of the UCNP constellation shown in Figure 3 can be characterized in terms of the number/dimension of nanoparticles per node and corresponding photon detection rate (encoded in false color in Figure 3B) that can be converted to photon emission rate (*P*_em_) per excitation intensity, which suffices to determine the UCNP conversion efficiency, *η*_uc_ = *P*_em_ */P*_abs_. The photon emission rate integrated over the node was obtained by reading out and converting the corresponding image pixels, since the epi-luminescence detection channel throughput, and the EMCCD spectral quantum efficiency versus the camera settings were calibrated (see Section 2.4.2, Figure S5 and Figure S4). The conversion efficiency was found by:
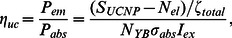
(3)where the quantities are explained in section 2.4.2.

The calculated *η*_uc_ of the single UCNP and two clusters (as identified in Figure 3) obtained by processing the image data is presented in Table 1 along with the relevant parameters. The excitation intensity corresponded to the saturation regime where the ensemble-averaged *η*_uc_ measured with the integrating sphere was nearly 2%. The calculated values of *η*_uc_ of the individual UCNP constellation nodes ranged between 1.2 and 2.0%, due to the variability in the host crystal composition and impurities. The independent *η*_uc_ measurements for the isolated and ensemble UCNPs exhibited excellent agreement, which ensured that the *η*_uc_ dependence on *I_ex_* and emission peak ratios hold for single UCNPs. Although the absolute *η*_uc_ of the single-UCNP was marginally lower than that of the ensemble-averaged UCNPs, it was adequately high to enable ultrasensitive imaging at the single-particle sensitivity level in biological scenarios, as addressed in the following section.

**Table 1:** Determination of the absolute *η*_uc_ using data from the calibrated epi-luminescence imaging of single and small clusters of UCNPs.

	*P_em_ [W]* (25%)	*N_Yb_ (10%)*	*σ_abs_ [cm^2^]* ^b)^	*I_ex_ [W/cm^2^](10%)*	*η_uc_ [%]*
Cluster 1^a)^	2.6×10^−14^	5.5×10^5^	1.0×10^−20^	2.6×10^2^	2.0±0.5
Cluster 2^a)^	2.2×10^−14^	6.1×10^5^	1.0×10^−20^	2.6×10^2^	1.3±0.3
Single^a)^	4.9×10^−15^	2.3×10^5^	1.0×10^−20^	2.6×10^2^	1.2±0.3

a) As identified in Figures 3B and 3C. ^b)^The Yb^3+^ absorption cross-section, as in Refs 15, 26. Unites are given in square brackets, percentage standard deviations are given in brackets, except for *η_uc_*.

### 3.2 Biomedical Imaging Applications

#### 3.2.1 Single-particle imaging through hemolyzed blood

**Figure 5:**
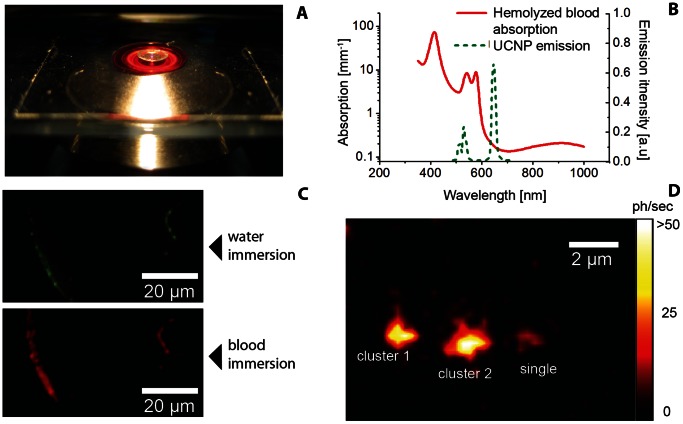
Epi-luminescence imaging of a single UCNP using a “blood-immersion” objective. (A) A photograph of the hemolyzed blood layer between the objective and cover slip. (B) Absorption spectrum of the hemolyzed blood (red solid curve) and UCNP emission spectrum (green dashed curve). (C) Low-magnification images of the UCNP sample recorded through the eyepiece port using the water- (top) and blood- (bottom) immersion objective. The dried UCNP colloid rims appeared green (top) and red (bottom) due to the green light absorption by blood. (D) Epi-luminescence microscopy image of the UCNP constellation identified in Figure 2C, imaged using the blood-immersion objective. The single UCNP is clearly observable, although blurred. The EMCCD camera settings and excitation parameters were equivalent to these of Figure 2. The pixel values were converted to photons/second (ph/s) and color-coded using the look-up bar in (D).

The feasibility of single-UCNP imaging in a biological environment was explored by obscuring the UCNP sample with a highly absorbing biological fluid, such as blood, with results presented in Figure 5. Whole blood (1 ml) was hemolyzed by replacing plasma with distilled water. After centrifuging the supernatant was used to replace the water between the objective and sample plane to create a “blood-immersion objective”, as shown in Figure 5A. The blood layer thickness was ∼250 µm. Hemoglobin, the oxygen-carrying protein in red blood cells, is the main absorber in blood and has a distinct absorption spectrum, as shown in Figure 5B. The UCNP emission spectrum is superimposed on the blood absorption spectrum to emphasize the high absorption in the green emission band and low absorption in the red emission band. This differential absorption by the blood altered the sample coloration, as shown in Figure 5C. A low-magnification image of the UCNP sample illuminated by the 978-nm laser was captured by a digital color camera through the eyepiece port of the epi-luminescence microscope, which reproduced the color perception by the human eye. The UCNP sample appeared green (Figure 5C, top panel), when using the standard water-immersion configuration of the objective lens, despite the prominent red band in the UCNP emission spectrum. This is explained by the higher spectral sensitivity of the eye to green color compared to that of red color (∼20 times [36]), which was also reproduced in the spectral sensitivity of the color camera. In case of the hemolyzed “blood-immersion objective”, however (Figure 5C, bottom panel), the green emitted light was largely absorbed by the hemoglobin, and the UCNP sample appeared red in the digital color image and to the human eye. Hence, the UCNP red emission and NIR excitation passed with small losses through the biological fluid, thus suggesting the feasibility of imaging single UCNPs. This ultrahigh-sensitivity imaging through an obscuring biological fluid was demonstrated, as shown in Figure 5D, where the previously identified single UCNP was clearly observable. The single-UCNP imaging has been reported using flying-spot [22], [24] and wide-field [23], [25] set ups, for exposed particles. To the best of our knowledge, this is the first demonstration of single-UCNP imaging in a biological environment, using a moderate excitation intensity (250 W/cm^2^) approaching the laser safety limits in a biological sample of realistic thickness. The demonstrated imaging through a 250-µm thick hemolyzed blood layer is, for example, favorably comparable with the diameters of blood vessels in the microcirculation, which can be found in proximity to organ surfaces [37].

These experimental results show the feasibility of optical imaging of single upconversion nanoparticles in biological fluids, in accordance with the assessment of the photophysical properties. These results constitute an experimental platform from which the detection limits of single-particle imaging in live skin is assessed theoretically, as presented in the next section.

#### 3.2.2 Modeling of single-particle imaging in skin

In comparison with conventional *in vivo* fluorescence-assisted imaging, UCNP-assisted imaging offers the advantage of reduced excitation/emission light losses and complete suppression of the optical background due to tissue autofluorescence [11]. The implication of this improvement is demonstrated by a simplified quantitative comparison of imaging in skin using fluorescein (FL) and UCNPs at the ultrahigh-sensitivity single-particle level. The choice for this representative visible organic fluorescent dye is dictated by its broad acceptance in the field of experimental skin research *in vivo* in animals and humans [38], as well as in diagnostic procedures in skin [39] and other organs [40]. In our model, a single emitter (FL and UCNP) is considered buried in skin at a depth *z*. The calculated single-emitter (*S*) and background (*B*) signals were compared in terms of their contrast (*S/B*) at different *z*. A confocal imaging setting is assumed in this model to reduce the adverse obscuring effect of out-of-focus signals that burden fluorescence-assisted imaging. The confocal imaging is known to improve the signal discrimination, such that the signals primarily originated from the focal volume at *z*.

The excitation power, 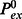 at the focal volume is attenuated according to Beer-Lambert’s law: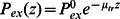, where *µ*_tr_ is the transport attenuation coefficient [mm^−1^] defined as a sum of the attenuation, *µ*_a_ and reduced scattering coefficient, *µ*_s_’ = (1-*g*)*µ*_s_, *µ*_s_ being the scattering coefficient, and *g* – the anisotropy factor. The attenuation is due to photon removal from the focusing path by absorption (*µ*_a_ ) and isotropic scattering (*µ*_s_’). *µ*_tr_ was computed in the visible and NIR spectral range using the reported values of *µ*_a_ and *µ*_s_’ for fresh Caucasian adult skin samples *in vitro*; while assuming a layered skin structure of epidermis, dermis and subcutaneous fat of corresponding thicknesses 80 µm, 400 µm and 520 µm, respectively [41]. The emitted power was calculated using 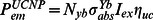
and 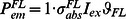 where *I*_ex_ – the excitation intensity at depth *z* calculated by dividing *P*_ex_ *(z)* by the excitation area. *Φ*_FL_ – the FL conversion efficiency was taken as 0.9 and 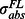 = 3.5×10^−16^ cm^2^ at *λ*_ex_ = 500 nm, [7] and 1 signifies a single FL emitter. 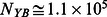
was calculated using the NaYF_4_ crystal lattice constants [27] and Na to Yb molar ratio [28] for a 70 nm particle, *η*_uc_ was obtained using the data plotted in Figure 2A at the corresponding values of *I*_ex_, and 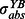 as listed in Table 1. The maximum permissible exposure (MPE) for skin is tabulated as 200 W/cm^2^ at 500 nm (FL) and 700 W/cm^2^ at 978 nm (UCNP) for a 1-ms exposure time [42]. The UCNP emission is attenuated along the return path through skin, which was accounted for using *µ*_tr_ of skin at the UCNP and FL emission wavelengths. The isotropic emission collection efficiency was determined by the objective lens acceptance angle (NA = 0.8). The photon conversion of the collected emission by the EMCCD camera (moderate EM gain, ×100) was computed using the calibration protocol presented earlier (Equation 3, Section 3.1.4.).

The single-UCNP imaging contrast was estimated using the background level set by the electronic noise, with dark and read noise components specified by the camera manufacturer (Andor), and shot noise calculated as 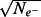
(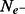, the photoelectron number before the EM-gain). The total noise was multiplied by the EM-characteristic multiplicative noise factor 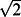. [43] The FL imaging background (*B*), however, had an additional autofluorescence-induced component. At *λ*_ex_ = 500 nm, the autofluorescence was mainly due to the abundant endogenous fluorophore in skin, flavin adenine dinucleotide (FAD), whose concentration in skin was reported to be ∼1 µg/mg [44]. ∼750 FAD molecules were situated in a 1-µm^3^ focal volume and contributed to *B*. In addition, the out-of-focus FAD-induced background signal was estimated following the model of confocal imaging by Magnor *et al*. for scattering media, assuming a homogeneous distribution of FAD in skin [45]. 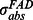
and *Φ*_FAD_ were taken as 6.0×10^−18^ cm^2^ at 500 nm and 0.033, respectively [46]. The FAD-induced electronic signal at the EMCCD camera was computed analogously to that of FL and UCNP, corrected for the spectral filtering of the FAD emission profile extending beyond the FL emission spectrum.

**Figure 6:**
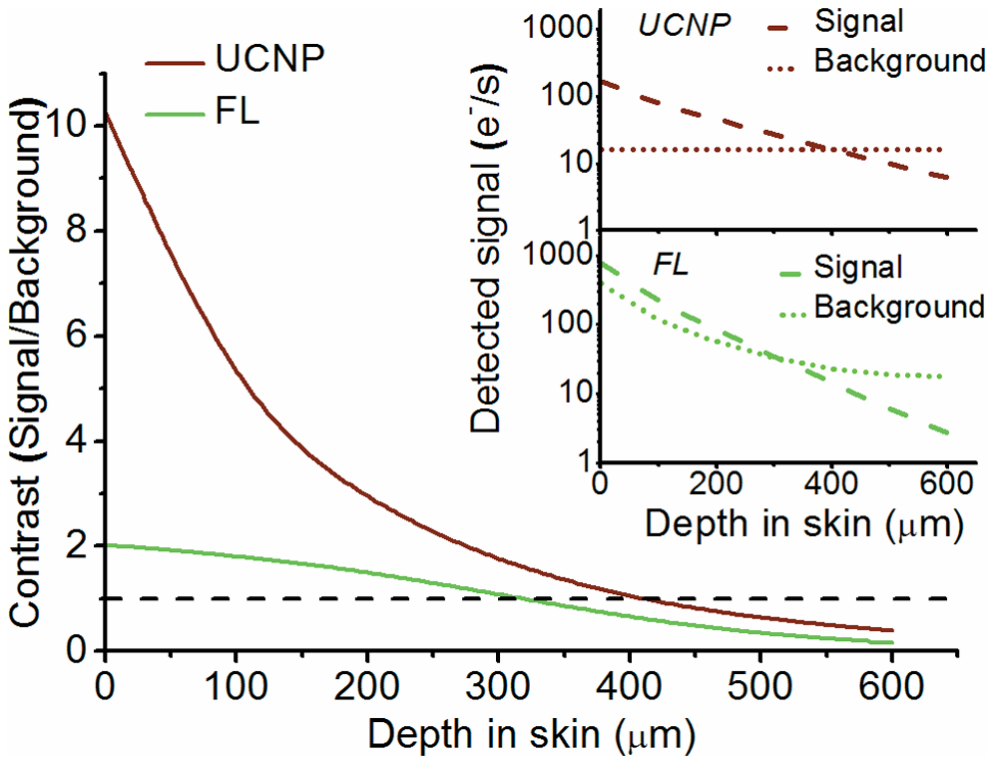
Theoretical estimation of single-emitter detection sensitivity in skin. A plot of the optical (confocal) detection contrasts of a single upconversion nanoparticle (UCNP, brown) and organic fluorescence dye (fluorescein, FC, green) versus their depth in skin, as modeled theoretically. The inset shows more detailed quantitative plots of the imaging signal (dashed) and background (dotted) of UCNP and FL versus depth in skin expressed in electrons per second (e^−^/s). The black dotted line demarcates the contrast value of 1. See text for details.

Figure 6 shows a plot of the calculated imaging contrast of a single emitter versus depth in skin tissue. The absolute luminescence signal *S* from FL is high at the shallow imaging depths (<300 µm) in comparison with that of UCNP, and so is *B* due to the autofluorescence by FAD (Figure 6, inset). The resultant contrast provided by FL is therefore very modest, with the highest value of 2 at the skin surface. Taking into consideration the non-uniformity of autofluorescence across the skin sample, single-FL imaging is at best problematic, if at all possible, particularly given the serious issue of the short lifetime of FL before undergoing photobleaching. In regard to *S/B* of the single UCNP, the relatively low *S* was offset by the extremely small *B,* almost invariable versus depth. The contrast is therefore, much higher, slowly decreasing from a value of 10 down to 3 over the 200-µm depth, which allows reliable detection of a single UCNP particle. Based on our model *S/B* reached a value of 1 at 400-µm depth in skin, however, due to a number of the simplifying assumptions made this is likely to be the best-case-scenario.

#### 3.2.3 Feasibility of single-particle imaging in biotissue

The remarkable progress in the synthesis of upconversion nanomaterials and demonstration of their unique luminescence properties have led to new possibilities in optical biomedical imaging using UCNPs. However, an uptake of this luminescent nanotechnology into cellular imaging is tempered by the existence of competitive luminescent nanomaterials that exhibit a high contrast against the dim autofluorescence background of cells (*e.g.* europium complexes, quantum dots). Nam et al. reported the imaging of UCNP-labeled live HeLa cells for 6 h using 980 nm excitation at I_ex_ = 3 kW/cm^2^, which allowed high-sensitivity intracellular imaging [47]. At these excitation intensities, *in vitro* single UCNP imaging in cells is feasible, as our results suggest. The scope of *in vivo* UCNP applications for optical deep tissue imaging of small lesions appears to be limited by the supralinear dependence of the upconversion luminescence on the excitation intensity, which is greatly diminished at *I*_ex_≪*I*_sat_ in deep (>1 cm) tissue layers. This confines the application niche of UCNP-based molecular probes to optical luminescent imaging in biological fluids, subsurface tissue layers and thick biological tissue slices. Within these domains, the UCNP-based molecular probes exhibit outstanding performance and provide unparalleled imaging capabilities, as was demonstrated and modeled in this paper (Single-particle imaging, Section 3.2, Figures 5 and 6). Such exceptional performance is achieved due to the unique photophysical properties of UCNP that allows evading the adverse effects of high absorption and autofluorescence of biological tissue, as long as the high excitation intensity is attainable by focusing.

The theoretical analysis highlights great promise for imaging single nanoparticles embedded in biological tissue, and provides a useful guidance to luminescent nanoparticle design (not necessarily limited to UCNP) and optical imaging modality. For example, an improved photochemical design of UCNPs, *e.g.* co-doping NaYF_4_ with Ytterbium and Thulium featuring an emission peak at 800 nm should further reduce the absorption by blood (Figure 5) and result in an increased imaging depth [48]. A promising recent result was the synthesis of 42-nm UCNPs with an exceptional high conversion efficiency at 800 nm (3.5%) [49], using core-shell synthesis to reduce surface quenching [50]. The maximum imaging depth (SNR >1) for the reported UCNP sample, with mean size 42 nm and the highest reported value of *η_uc_* = 3.5%, was found to be 450 µm (see Figure S7).

There are several reports demonstrating UCNP-assisted imaging depths of 2 cm [13] and 3.2 cm [51] in live mice and fat tissue respectively, using a large number of UCNPs. In this paper, the absolute detection sensitivity of single UCNP imaging *in vivo* is addressed, which can readily be extended to an ensemble of UCNPs.

At the same time, our presented optical imaging model was oversimplified. The confocal flying-spot scanning modality is impractically slow due the long (sub-milliseconds) UCNP luminescence lifetime, which requires to the dwell time to be set to ∼500 µs per pixel to avoid smearing of the emission light onto adjacent pixels due to the UCNP after-glow [21]. On the other hand, the wide-field epi-luminescence modality suffers from the overshadowing of small single-UCNP signals by large UCNP clusters, so that on some occasions, we had to reduce the field diaphragm of the epi-luminescence microscope to collapse the field-of-view to a diameter of ∼20 µm. The wide-field configuration was not efficient in suppressing out-of-focus signals, which precluded the use of whole blood due to scattering, and required blood hemolyzation. This could possibly be prevented by optical clearing mechanisms that result in a reduced attenuation coefficient of whole blood [52]. Lastly, the NIR irradiation dose (250 W/cm^2^ CW) still exceeded the maximum permissible exposure for skin. Although excitation at 980 nm at a ten-fold greater intensity has been shown to be tolerable to live cells for 6 h *in vitro* [47], continuous *in vivo* imaging at the excitation intensity as low as 0.5 W/cm^2^ caused significant skin damage to a mouse [53], due to the high water absorption in the live animal. Shifting the excitation wavelength from 980 to 915 nm was suggested to reduce water absorption. A pulsed laser excitation regime delivering high energy per pulse at a low repetition rate represents another possible solution to maintain acceptable imaging contrast within the laser safety limits. Therefore, advanced optical imaging – possibly, a hybrid of the wide-field epi-luminescence and confocal modalities – is required to harness the UCNP specificity and uniqueness.

There are a number of interesting prospects to employ UCNPs as molecular probes in tissues sites accessible to ballistic photons including skin, the superficial microcirculation and sub-surface lesions for luminescence-guided surgery. Recent reports demonstrate that imaging of low amounts of UCNP-labeled stem cells is feasible: as few as ∼10 labeled cells were subcutaneously detected in nude mice and *in vivo* detection limits for UCNP-based imaging were an order of magnitude lower in comparison with QDs [54], [55]. UCNPs conjugated to a cancer-targeting peptide, for example somatostatin [56], [57] can facilitate the diagnosis of skin cancer [58]; detection of circulating tumor cells in subcutaneous blood vessels, or the sensitive guidance to tumor sites by receptor-targeted optical imaging [59]. Recently, we have successfully conjugated UCNPs with scFv4D5 mini-antibodies raised against the human epidermal growth factor receptor (HER2/neu) overexpressed in e.g. breast adenocarcinoma cells and demonstrated high-specific binding *in vitro* (unpublished results). Delivery of conjugated UCNPs to tumors *in vivo* poses new challenges. The primary delivery mechanism of intravenous injected nanoparticles is thought to be the enhanced permeability and retention effect, caused by leaky blood vessel walls in tumor tissue. Subsequent nanoparticle diffusion into the tumor core occurs via intercellular or intracellular routes, where the latter mediated by endocytosis is more likely than the former due to the large nanoparticle size compared to the intercellular space [47], [60].

As a last example, UCNP-facilitated ion sensing by combining the optical properties of ion sensing chromophores/fluorophores with UCNP optical characteristics seems to be promising [61].

### Conclusion

The adoption of upconversion nanomaterials in optical biomedical imaging demands specification of application areas where the UCNP merits are critical while the limitations are tolerable, and where the cutting-edge sensitivity required for UCNP-assisted imaging performance is achievable. We examined this ultrahigh-sensitivity optical imaging scenario by performing imaging and characterization of a single UCNP in a biological environment represented by hemolyzed blood. In particular, the key UCNP parameters of absolute conversion efficiency and spectral emission were measured in individual and ensemble particles and found comparable. These experimental results were utilized in an idealized theoretical model, which was believed to aid identification of application areas of extreme sensitivity in challenging *in vivo* environments, including biological liquids, subsurface layers and thick tissue slices. Specifically, our theoretical skin imaging model showed that UCNP imaging had superior contrast over that of conventional fluorescent dyes. The background-free imaging of single upconversion nanoparticles at depths up to 400 µm in skin was found feasible. Therefore, the application scope of carefully tailored UCNP-based molecular probes is significant, including luminescence-guided surgery and ultrahigh-sensitivity bioassays in unprocessed biological fluids.

### Supporting Information

Supporting information is available online and includes additional information regarding the quantitative imaging method and characterization of the UCNPs.

## Supporting Information

Figure S1 **Set-up and Köhler ray diagram.** The set-up consisted of a wide-field inverted epi-fluorescence microscope (Olympus IX70) equipped with a water-immersion objective (40×, NA 1.15, Olympus). The laser illumination was guided to the sample plane using a modified illumination path replacing the default Mercury/Xenon arc lamp housing. To achieve homogeneous illumination at the sample plane and to have precise control over the field-of-view, a modified Köhler illumination scheme was built as is shown in panel (a). The two Köhler illumination ray diagrams illustrating the homogeneous illumination light ray path and the image forming light ray path are shown in panels (k-1) and (k-2) respectively. The conjugate planes are explained in the legend. In (k-1) a collimated laser beam filled the adjustable iris located at the back focal plane of the field lens, which was, in turn, one focal distance away from the back focal plane of the objective resulting in even and collimated sample illumination. In (k-2) the image forming function of the Köhler scheme is depicted: the image of the iris located at the focal point of the field lens resulted in the formation of its image at the objective focal plane. The magnification of the iris image was the ratio between the focal lengths of the objective and the field lens (∼28× in this setup). A zero-aperture iris was used to control the illumination area. The other components of the set-up are an electron-multiplying CCD (EMCCD) camera (Andor iXon DU-885) and an acousto-optic tunable filter (AOTF) (LSi-300 Hyperspectral Imaging System, Gooch and Housego) mounted to the left side port of the microscope; excitation and emission filters; a 978 nm laser (LD980-01CW, CXCH-Photonics) and an mechanical dither to average out speckles. These components are further discussed in the main paper.
(TIF)

Figure S2 **Determination of background excitation light.** To estimate the amount of excitation light bleeding through the emission filter set and reaching the EMCCD camera, we imaged a TEM-grid with reference material (TiO_2_) and obtained the pixel values on a line through the field-of-view on the image. The graph shows the pixel values in counts for each pixel along the line (pixel number) for the reference TEM-grid and for a TEM-grid with UCNP sample deposited. As can be seen no elevation of pixel value was observed for the reference image at the field-of-view, whereas the sample image pixel values increased at the focus spot due to detection of upconverted light. The detected signal was nonzero due to addition of a pixel base level and (low) values for read and dark noise. We can conclude that virtually no excitation light reached the detector.
(TIF)

Figure S3 **Excitation intensity dependence of the green and red emission bands.** (A) Ensemble emission spectra of UCNP powder were measured in transmission using a customized glass sample holder and calibrated spectrometer for several excitation intensities at excitation wavelength 978 nm (see section 2.3). The spectra were normalized at the 540 nm peak and are presented with an offset for clarity. (B) Integrating the spectra of (A) over the green and red emission band resulted in a green-to-red-ratio (GRR) dependence on excitation intensity. In (C), the emitted power dependence on excitation intensity in the green and red emission band is plotted on a double-logarithmic scale that showed a linear slope until emission saturation is reached around 150 W/cm^2^. The slope is 1.9 for red emission and 1.5 for green emission, confirming a nonlinear emission process that involves at least 2 photons. Panel (D) shows the conversion efficiency (*η*_uc_) dependence of the green and the red emission bands on excitation intensity.
(TIF)

Figure S4 **Calibrated detector response for single UCNP immersed in water or blood.** Using the emission spectrum of the single UCNP (Figure 4A) and Equation 2 the spectral calibration coefficient in counts/W/s for each wavelength can be calculated assuming *T = *1 s, *EM gain = *1, *S_ccd_* = 0.89 e^−/^count and *N_ph_* the number of photons per wavelength per UCNP emission power of 1 Watt. Data points are connected by a solid line. In case of the hemolyzed blood-immersion objective the spectral detector response is reduced due to absorption of light by hemoglobin. The absorption is high for the green emission band and low for the red emission band. Integrating over all wavelengths results in total counts/W/s for UCNP emission, referred to as ζ_total_.
(TIF)

Figure S5 **Spectral throughput of inverted epi-luminescence microscopy system.** The absolute throughput, *ξ_optics_,* defined as *signal_sensor-plane_/signal_sample_*_-plane,_ of the microscope imaging system was obtained by imaging an optical fiber (multimode, 100 µm core, NA 0.22) placed at the sample plane of the microscope. The output power of the fiber was measured using a calibrated power meter (Thorlabs). Using the camera settings and sensitivity specifications the power detected by the sensor can be calculated from the image pixel values, and subsequently the throughput can be obtained. This was done for several wavelengths and spectrally interpolated using a white light source (Ocean Optics LS-1, tungsten halogen), as plotted in the graph. The discontinuity around 510 nm was due to the dichroic beam splitter with a cut-off wavelength at 511 nm. Assuming isotropic emission of upconverted light by the UCNPs, the actual throughput of the system needed to be corrected for the acceptance angle of the microscope objective, a factor 1/3 for NA = 1.15 (not shown).
(TIF)

Figure S6 **TEM images with different magnifications of in-house synthesized UCNPs.**
(TIF)

Figure S7 **Theoretical estimation of single UCNP detection sensitivity in skin, a comparison between in-house synthesized and state-of-the-art UCNPs.** The highest conversion efficiency of 3.5% was reported for Yb,Tm-co-doped core-shell UCNPs (ref 49). Using our model described in Section 3.2.2, we calculated the imaging signal contrast and plotted the result (blue dot-dashed line) together with the previous results for fluorescein and the 70-nm UCNP. The 42-nm 3.5%-UCNP has a reduced absorption of excitation light due to the smaller size of the particle which reduced the imaging contrast, but the higher *η_uc_* and the reduced transport attenuation coefficient of skin for the luminescence signal resulted in a higher contrast at increasing depth. The maximum imaging depth in skin is 450 µm, with contrast 1.
(TIF)
