# Supplementary figures and images for: Quantitative Imaging of Single Upconversion Nanoparticles in Biological Tissue
Source: PLoS One. 2013 May 14;8(5):e63292. doi: 10.1371/journal.pone.0063292 (PMC3653952; doi:10.1371/journal.pone.0063292)

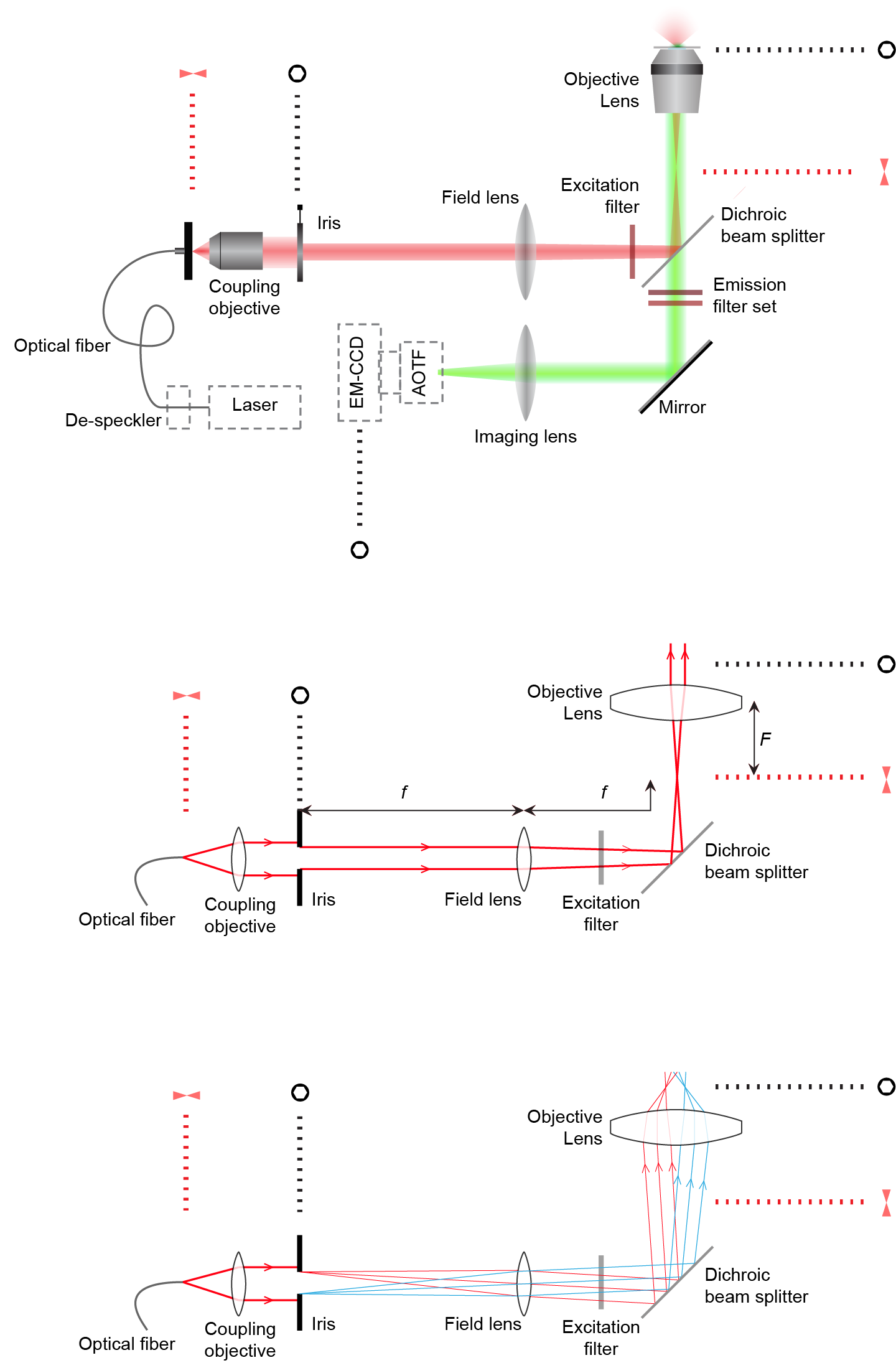

Supplement: Figure S1 — Set-up and Köhler ray diagram. The set-up consisted of a wide-field inverted epi-fluorescence microscope (Olympus IX70) equipped with a water-immersion objective (40×, NA 1.15, Olympus). The laser illumination was guided to the sample plane using a modified illumination path replacing the default Mercury/Xenon arc lamp housing. To achieve homogeneous illumination at the sample plane and to have precise control over the field-of-view, a modified Köhler illumination scheme was built as is shown in panel (a). The two Köhler illumination ray diagrams illustrating the homogeneous illumination light ray path and the image forming light ray path are shown in panels (k-1) and (k-2) respectively. The conjugate planes are explained in the legend. In (k-1) a collimated laser beam filled the adjustable iris located at the back focal plane of the field lens, which was, in turn, one focal distance away from the back focal plane of the objective resulting in even and collimated sample illumination. In (k-2) the image forming function of the Köhler scheme is depicted: the image of the iris located at the focal point of the field lens resulted in the formation of its image at the objective focal plane. The magnification of the iris image was the ratio between the focal lengths of the objective and the field lens (∼28× in this setup). A zero-aperture iris was used to control the illumination area. The other components of the set-up are an electron-multiplying CCD (EMCCD) camera (Andor iXon DU-885) and an acousto-optic tunable filter (AOTF) (LSi-300 Hyperspectral Imaging System, Gooch and Housego) mounted to the left side port of the microscope; excitation and emission filters; a 978 nm laser (LD980-01CW, CXCH-Photonics) and an mechanical dither to average out speckles. These components are further discussed in the main paper. (TIF) [file pone.0063292.s001.tif]

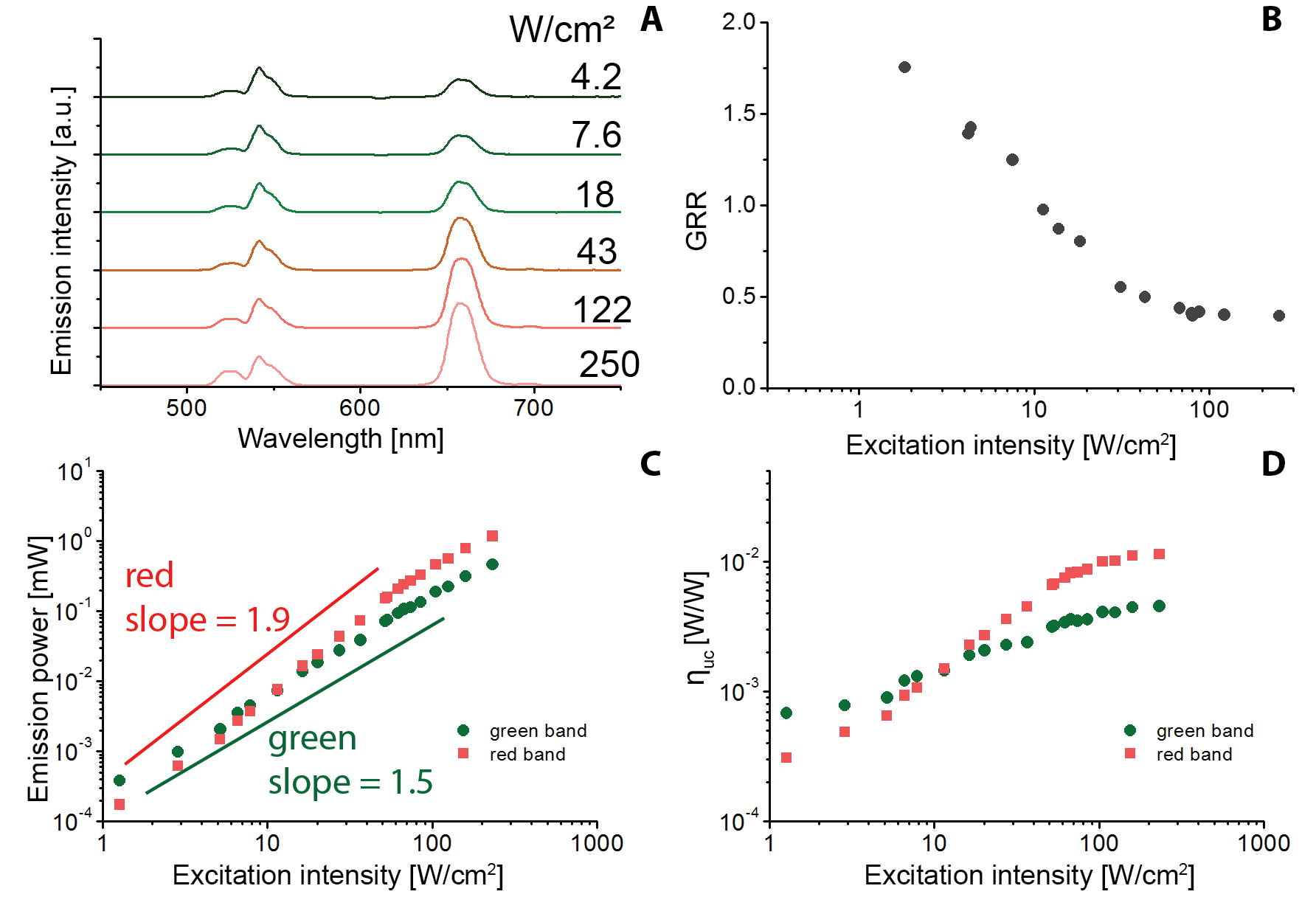

Supplement: Figure S3 — Excitation intensity dependence of the green and red emission bands. (A) Ensemble emission spectra of UCNP powder were measured in transmission using a customized glass sample holder and calibrated spectrometer for several excitation intensities at excitation wavelength 978 nm (see section 2.3). The spectra were normalized at the 540 nm peak and are presented with an offset for clarity. (B) Integrating the spectra of (A) over the green and red emission band resulted in a green-to-red-ratio (GRR) dependence on excitation intensity. In (C), the emitted power dependence on excitation intensity in the green and red emission band is plotted on a double-logarithmic scale that showed a linear slope until emission saturation is reached around 150 W/cm2. The slope is 1.9 for red emission and 1.5 for green emission, confirming a nonlinear emission process that involves at least 2 photons. Panel (D) shows the conversion efficiency (η uc) dependence of the green and the red emission bands on excitation intensity. (TIF) [file pone.0063292.s003.tif]

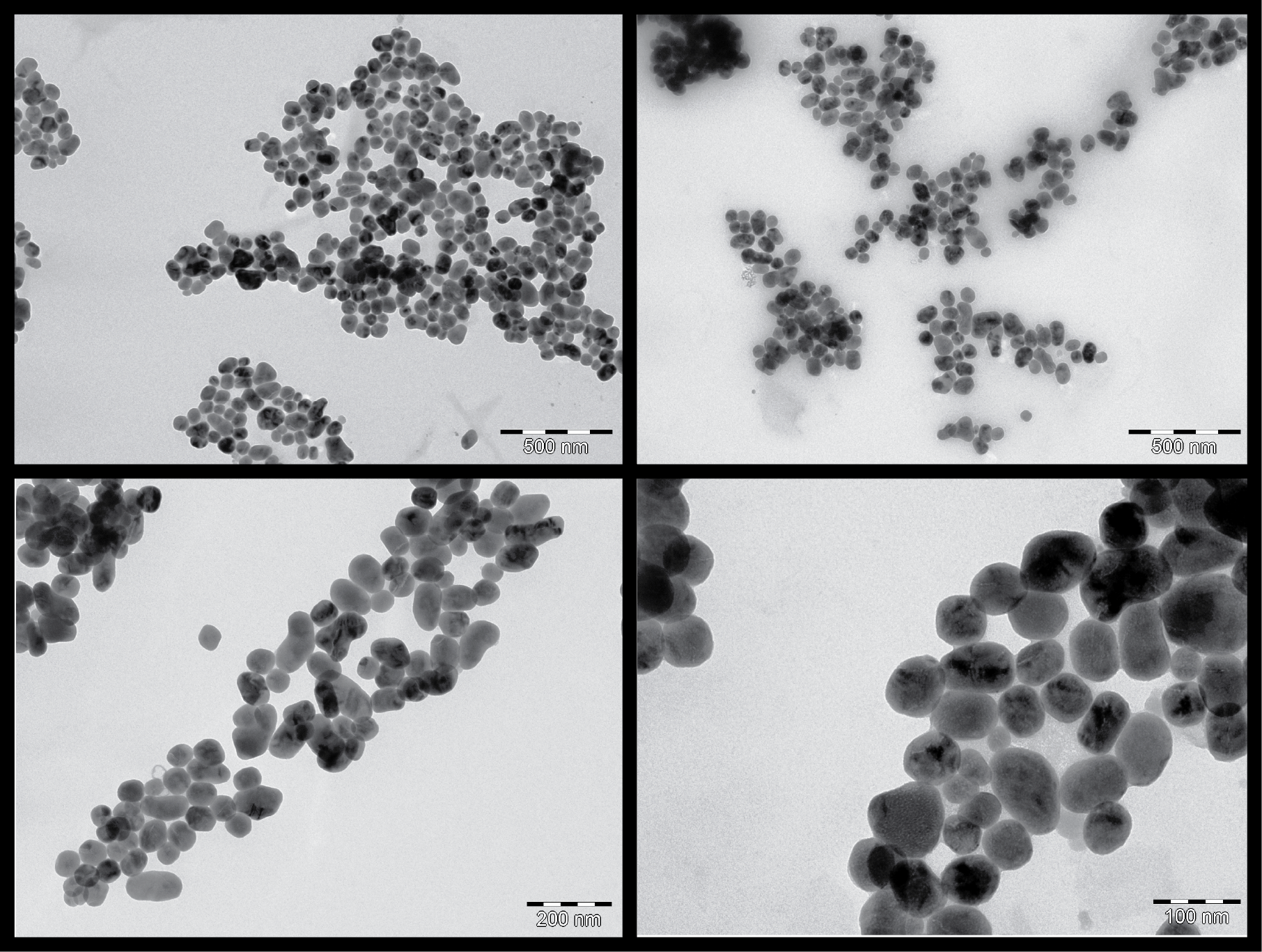

Supplement: Figure S6 — TEM images with different magnifications of in-house synthesized UCNPs. (TIF) [file pone.0063292.s006.tif]
